# Leveraging feature extraction and risk-based clustering for advanced fault diagnosis in equipment

**DOI:** 10.1371/journal.pone.0314931

**Published:** 2024-12-30

**Authors:** Hyeonbin Ji, Ingeun Hwang, Junghwon Kim, Suan Lee, Wookey Lee

**Affiliations:** 1 Department of Industrial Engineering, Inha University, Incheon, South Korea; 2 School of Computer Science, Semyung University, Jecheon, Chungchungbuk-do, South Korea; University of Southern California, UNITED STATES OF AMERICA

## Abstract

In the contemporary manufacturing landscape, the advent of artificial intelligence and big data analytics has been a game-changer in enhancing product quality. Despite these advancements, their application in diagnosing failure probability and risk remains underexplored. The current practice of failure risk diagnosis is impeded by the manual intervention of managers, leading to varying evaluations for identical products or similar facilities. This study aims to bridge this gap by implementing advanced data analysis techniques on maintenance data from an aluminum extruder. We have employed text embedding, dimensionality reduction, and feature extraction methods, integrating the K-means algorithm with the Silhouette Score for risk level classification. Our findings reveal that the combination of Word2Vec for embedding and Contractive Auto Encoder for dimensionality reduction and feature extraction yields high-performance results. The optimal cluster count, identified as three, achieved the highest Silhouette Score. Statistical analysis using one-way ANOVA confirmed the significance of these findings with a p-value of 5.3213 × *e*^−6^, well within the 5% significance threshold. Furthermore, this study utilized BERTopic for topic modeling to extract principal topics from each cluster, facilitating an in-depth analysis of the clusters in relation to the extruder’s characteristics. The outcome of this research offers a novel methodology for facility managers to objectively diagnose equipment failures. By minimizing subjective judgment, this approach is poised to significantly enhance the efficacy of quality assurance systems in manufacturing, leveraging the robust capabilities of artificial intelligence.

## 1 Introduction

The assessment of risks associated with facilities is a critical endeavor aimed at ensuring their safety and mitigating accidents. To this end, Failure Modes and Effects Analysis (FMEA) has become a cornerstone in the arsenal of many businesses and manufacturing units, serving as a preventative mechanism against system, design, and process failures [1]. FMEA employs the Risk Priority Number (RPN), a quantifier of risk derived from the product of Occurrence (O), Severity (S), and Detection (D) factors, to prioritize and manage potential risks.

Despite the utility of FMEA in enhancing system reliability, its methodology for computing RPN has faced scrutiny. This critique stems from issues such as the production of identical RPNs for varied parameter values, indicating redundancy [2], an over-sensitivity to minor modifications, and a lack of adequate scaling to reflect the true significance of the O, S, and D components [3]. A fundamental flaw lies in the subjective interpretation of these factors, leading to a lack of objective, numerical clarity in risk assessment. The reliance on managerial judgment introduces a layer of uncertainty, undermining the consistency and reliability of RPN evaluations [4]. Addressing these deficiencies and the challenge of subjective judgment remains a pressing need [5].

This paper addresses the aforementioned limitations by proposing a quantitative approach to risk level assessment through the analysis of facility maintenance data and reports. Specifically, it utilizes data from an aluminum extrusion and casting company collected between August to December 2014 and January to November 2016. This study leverages the preprocessing of textual maintenance records, including failure sites and descriptions, applying advanced embedding techniques like Word2Vec and TF-IDF to craft embedding vectors. Subsequent application of dimensionality reduction and feature extraction through models such as Truncated SVD and AutoEncoders facilitates a nuanced analysis. The determination of an optimal cluster count, via Silhouette Score, and the categorization of risk levels using K-means clustering, underscore a methodological pivot towards excluding subjective biases in risk level. This objective classification is further refined by attributing risk levels based on failure intensity rates, thus ensuring a methodologically sound, manager-independent risk assessment process.

Furthermore, the study tackles the challenge of discerning specific failure modes within a voluminous array of documents. By extracting topics from a comprehensive document set and employing the BERTopic algorithm for topic modeling, this research elucidates the distinct impacts of risk levels on system and facility operations. Through this innovative approach, the study aims to offer a clearer, more objective lens through which to view facility risk levels, thereby enhancing the precision and reliability of risk management practices.

Based on the textual data, the study categorized quantitative risk levels with statistical significance, extracted key topics for each rating, and derived site-specific correlations. The contributions are as follows:

- Quantitative risk classification and statistical significance verification based on textual data and failure intensity rates from equipment failure reports.
- Compared to the existing method, which heavily relies on manager’s subjectivity, the risk classification completely excludes manager’s subjectivity.
- Based on the actual process data, we explained the main failure areas, relationships, and characteristics of each risk level.

This study is organized as follows. Section 2 provides a general description of the parts of the extruder, and Section 3 introduces prior research on risk level and topic modeling. Section 4 describes the theoretical background of the methodology and models used in the thesis. Section 5 describes the preprocessing of the equipment failure reports and the columns we added. Section 6 describes the functions and hyperparameter settings for the analysis. And we present the visualization and organization of the analysis results and the BERTopic results by clusters based on the results to analyze the failure contents of aluminum extruders. Finally, Section 7 concludes with a discussion and conclusion. Through this study, we aim to improve the existing qualitative risk classification method based on managers’ opinions and identify the causes of major failures in the field of aluminum extrusion facilities.

## 2 Background

### 2.1 Extruders

Extruders play a pivotal role in the metal and alloy processing industry, serving as fundamental machinery that shapes materials into specific profiles. These extruder plants are classified into two primary categories: single and multiple extruders, each tailored to different production needs. The design and layout of extruder plants are critical, involving a detailed analysis of the extruder’s size, shape, heating mechanisms, lubrication systems, and control devices. This meticulous planning is essential for optimizing the extruder’s performance and efficiency, as improper layouts can result in significant energy wastage and subpar product quality.

**Fig 1:**
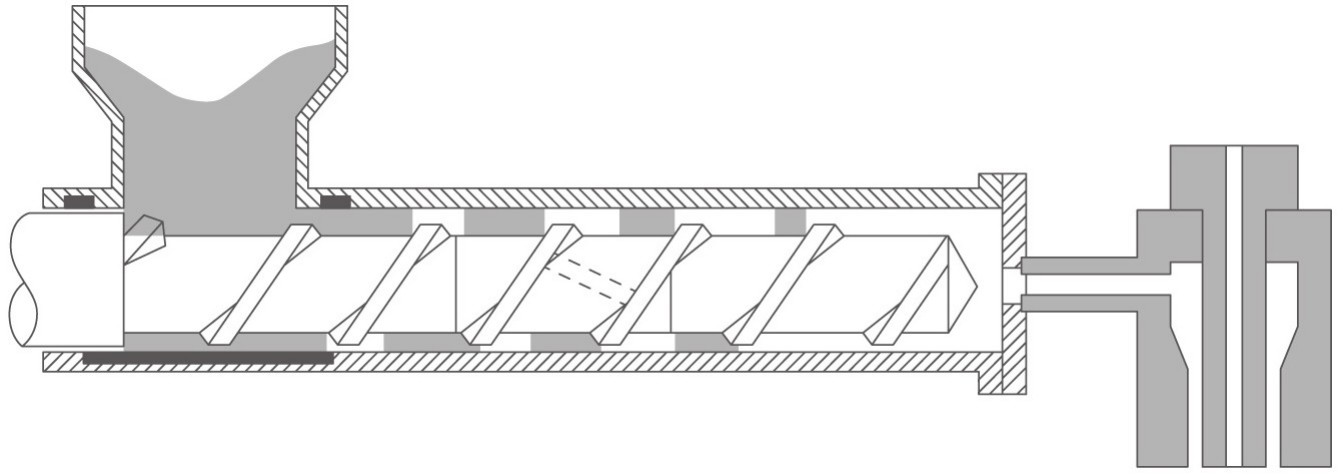
Structure of an extruder.

The components of an extruder, as depicted in Fig 1, such as dies, rams, pullers, screws, and bearings, play a crucial role in determining the machine’s processing capabilities, operational speed, and the quality of the output. Additionally, extruders are equipped with various heating devices—gas, electric, or fluid—to accommodate specific material processing needs. The choice of heating technology is based on the extruder’s requirements and the properties of the materials being processed. In terms of processing techniques, extruders utilize a range of methods including stretching, pulling, quenching, stress relieving, and recovery, each selected based on the specific needs of the material and the desired characteristics of the final product.

To further enhance productivity and efficiency, modern extruder plants incorporate advanced features such as automation, precise heating control, sophisticated lubrication systems, and quality inspection protocols. These innovations facilitate the production of higher-quality products and have cemented extruders’ role in industries such as automotive, aerospace, construction, and electronics, significantly contributing to the enhancement of product quality and manufacturing productivity.

### 2.2 Pullers

**Fig 2:**
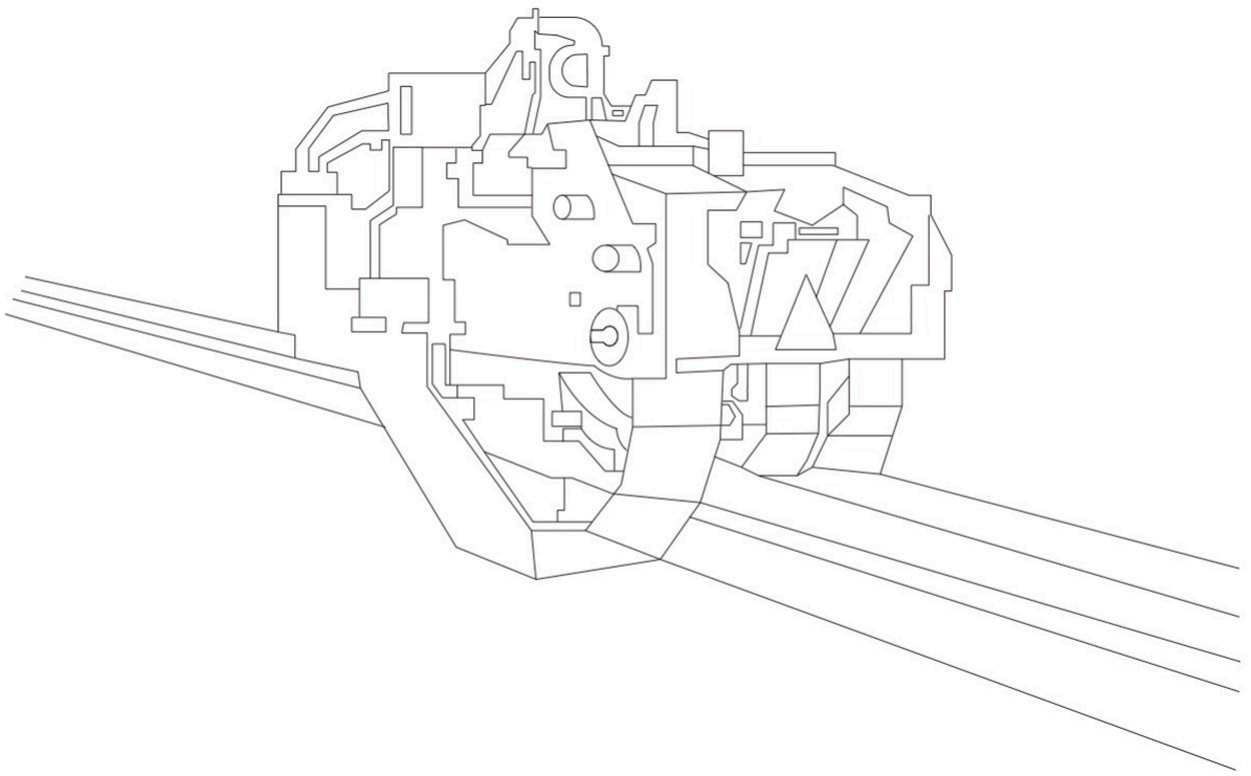
Structure of a puller.

Pullers, as depicted in Fig 2, as integral components of the extrusion process, are positioned downstream of the die to extract the extruded material. Typically comprising two sets of rollers, pullers not only facilitate mechanical movement but also regulate the speed and tension of the extruded material, ensuring consistent quality and preventing distortion. Moreover, pullers often play a role in the cooling process, using tank or other mediums to control the temperature of the material, thus minimizing potential deformation.

### 2.3 Backside

**Fig 3:**
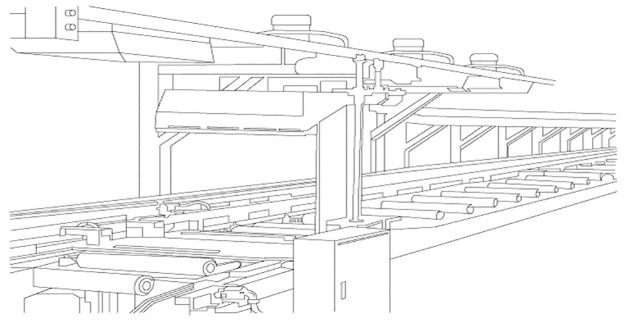
Structure of a backside.

The backside process, or the backside operation, encompasses a series of critical steps designed to refine the extruded product into its final form, as shown in Fig 3. This includes cooling, cutting, machining, and finishing processes, each integral to maintaining the product’s integrity and performance. Proper cooling is particularly vital to prevent internal stresses that can compromise product durability. Precision in cutting and machining is also crucial, as these processes directly affect the product’s dimensions and surface quality, underscoring the importance of rigorous quality control throughout the backside phase.

### 2.4 Hot saw

**Fig 4:**
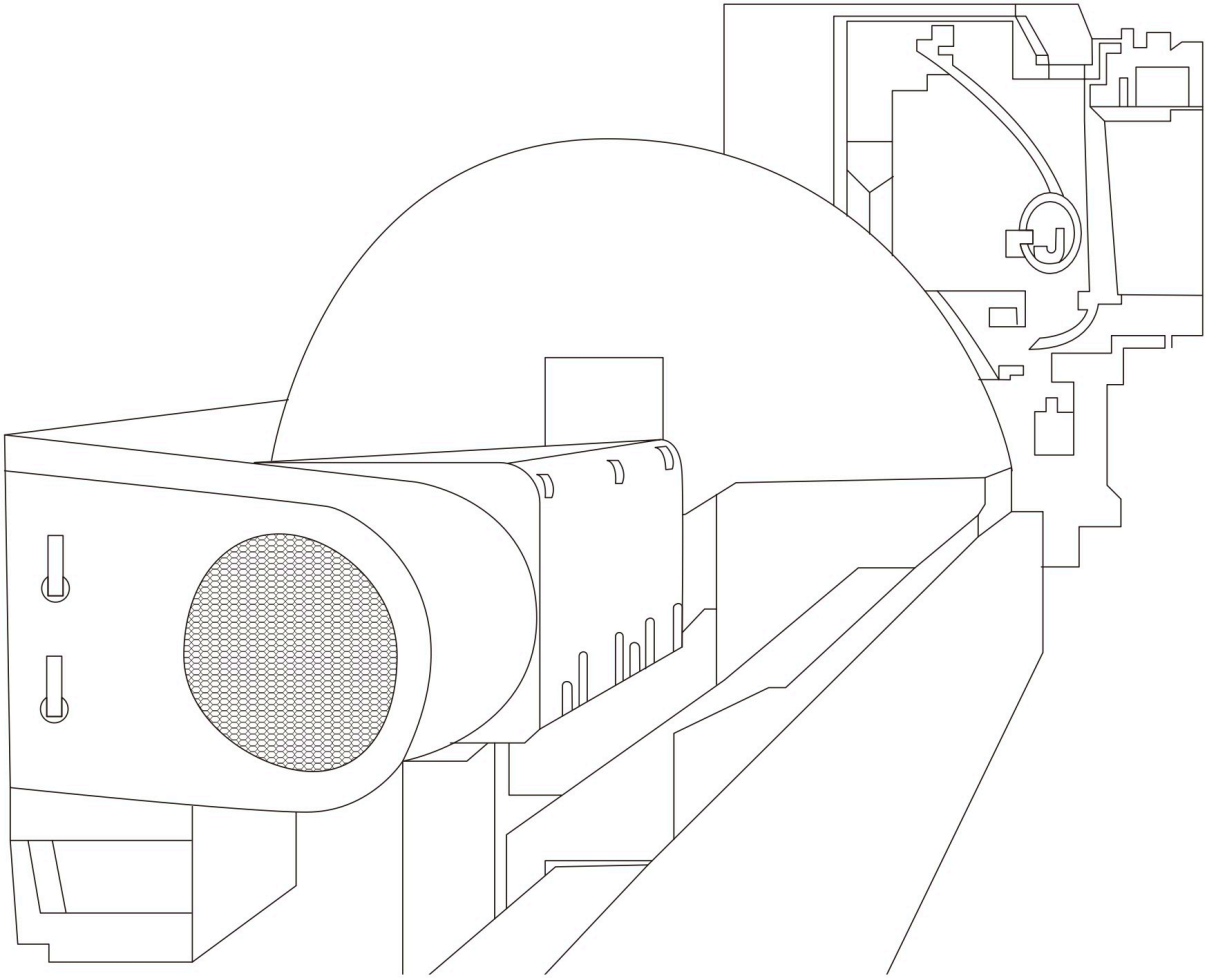
Structure of a hot saw.

Hot saw represents a specific cutting technique employed in the processing of metal products backside, as shown in Fig 4. This method, which involves cutting at elevated temperatures, is crucial for achieving precise lengths before the product cools down. Hot sawing’s efficiency, especially with the advent of high-speed cutting machines, ensures that the extruded metal parts conform exactly to the specifications of the final product, highlighting its indispensable role in the extrusion process.

## 3 Related works

### 3.1 Risk level research

The domain of risk level has witnessed considerable development across various sectors, emphasizing the significance of accurate and reliable risk assessment mechanisms in enhancing maintenance management and operational safety. Among these methodologies, Failure Modes and Effects Analysis (FMEA) stands out as a fundamental tool for risk identification and assessment [6]. Despite its widespread application, FMEA has been subject to critique due to its methodological limitations, including the occurrence of redundant Risk Priority Number (RPN) values that fail to distinguish between different risk levels adequately [2]. Critics also highlight issues such as FMEA’s excessive sensitivity to minor variations and its RPN scaling’s failure to appropriately weigh the significance of each risk factor (Occurrence, Severity, Detection) [3, 7]. Such limitations underscore the challenges in relying on managerial judgment, pointing to an urgent need for addressing these methodological flaws and the inherent subjectivity and uncertainty [4].

Various studies have sought to advance FMEA and address these challenges by integrating advanced analytical techniques. FMEA-QFD approach has been applied to logistics processes, highlighting key risk factors and defining corrective actions to improve warehouse and transport operations [8]. A data-driven FMEA method has been developed to address subjectivity and risk prioritization issues by incorporating objective data-based risk factors and advanced criteria ranking techniques [9]. Multi-Criteria Decision Making (MCDM) emerges as a promising approach, offering a more evaluation by considering multiple criteria simultaneously, thereby facilitating more precise and rational decision-making processes. Distance-based methods are commonly employed in MCDM for FMEA, as they assess the importance of risk factors and rank failure modes using mathematical formulas, ensuring a more objective prioritization process [10]. However, MCDM’s reliance on multiple criteria also introduces the risk of subjectivity, potentially complicating the identification of optimal solutions [6]. The Fuzzy Rule-Based System, leveraging fuzzy logic to manage uncertain and ambiguous information, presents a flexible solution through its rule-based operation. Despite its adaptability, the complexity and user-defined nature of its rules pose significant challenges, particularly in terms of maintainability within equipment applications [11]. There are also studies that integrate Fuzzy, FMEA, and VIKOR methodologies to address risk assessment challenges and enhance decision-making processes [12].

Grey Relation Analysis (GRA), renowned for its ability to elucidate the relationships among variables in complex systems, offers valuable insights, especially in scenarios where traditional modeling techniques fall short. Despite its effectiveness, GRA alone often struggles with the complexities of non-linear interactions among variables, necessitating hybrid approaches for improved performance [10]. The application of GRA in high-dimensional models remains nascent, with further research needed to establish robust validation methods and quantitative criteria [13]. Combining fuzzy logic’s strengths in handling uncertainty with GRA’s analytical precision, Fuzzy-Grey Relation Analysis emerges as an effective tool for tackling decision-making under uncertainty. However, its application, particularly in machine learning contexts, is hindered by the risk of overfitting, which can severely impair the model’s generalizability to new data [14].

### 3.2 Topic modeling research

In the realm of data analytics, extracting meaningful insights from voluminous datasets is paramount. This is particularly true for text analytics, where identifying and analyzing text topics can unveil underlying patterns and themes. Topic modeling, a statistical technique for discovering latent variables within large text corpora, has gained traction across diverse fields [15]. Techniques such as Latent Dirichlet Allocation (LDA) [16], Latent Semantic Analysis (LSA) [17], and BERTopic [18] are among the various models employed to distill essential features from text data, proving invaluable in numerous industrial applications.

Innovative applications of topic modeling include Sun, Lijun, and Yafeng Yin’s analysis of 17,163 journal articles using LDA to explore transportation research themes, shedding light on the evolution of research topics across journals, years, and geographic regions [19]. Ana Catarina Calheiros applied topic modeling to 401 review comments from 3,179 reservations, categorizing consumer sentiments in online hotel reviews to identify opportunities for enhancing customer experiences [20]. Wayne Xin Zhao’s study utilized the Twitter-LDA model to compare topics from Twitter with those in the New York Times, analyzing aspects such as tweet-to-retweet ratios and topic categories [21].

Within manufacturing, Hui Xiong’s examination of 82,248 article abstracts through an LDA model provided insights into topical trends over time, illustrating the dynamic nature of manufacturing research [22]. Similarly, Yang Hyun-Lim and colleagues’ quantitative analysis of smart factory-related research using topic modeling revealed distinct trends between Korean and international research landscapes, highlighting the global interest in smart manufacturing technologies [23].

## 4 Methodology and theoretical background

**Fig 5:**
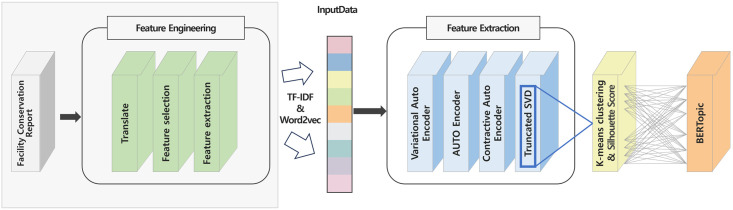
Overview of the proposed method.

The method proposed in this study is shown in Fig 5. We analyzed the equipment maintenance data of an aluminum extrusion and casting company from August to December 2014 and January to November 2016. The data consisted of reports of the equipment’s exhalation, failure date by part, failure content, and handling. We preprocessed the textual data of the failure site and failure content in the actual report, and then applied various embedding methods such as Word2Vec and TF-IDF to generate embedding vectors. We then applied dimensionality reduction and feature extraction models such as Truncated SVD, AutoEncoder, CAE, and VAE to reconstruct the embedding vector. After calculating the optimal number of clusters based on Silhouette Score, we classified the risk levels using K-means clustering technique. Each cluster was then assigned a risk level based on the reported failure intensity rate and checked for statistical significance. Due to the large number of documents within each risk level, it was determined that it would be difficult to analyze each document manually to identify the risk level (failure mode). To solve this problem, we leveraged the BERTopic topic modeling algorithm to extract topics from the large document set and identify the impact of each risk level on the system and equipment.

### 4.1 Text embedding

In the realm of natural language processing (NLP), text embedding is a foundational technique that translates human language into a numerical form that machines can interpret. This involves converting words or sentences into vectors within a high-dimensional space, where linguistic similarities are mirrored by spatial proximity. This study employs Word2Vec and TF-IDF as primary text embedding algorithms, facilitating the transformation of text into distributed representations. Such embeddings are crucial for subsequent clustering and classification tasks, enabling the effective analysis of texts with analogous meanings.

#### 4.1.1 Word2Vec

Word2Vec represents words through dense vectors in a low-dimensional space, offering a significant advantage over traditional sparse representations like one-hot encoding. This method captures semantic relationships between words, allowing for operations that can discern linguistic analogies, such as vector(“King”)-vector(“Man”)+vector(“Woman”) approximating the vector for “Queen” [24]. Word2Vec operates through simple neural networks without activation functions, employing either Continuous Bag of Words (CBoW) or Skip-gram models for training. This study adopts the CBoW model for its efficiency in leveraging context to predict word meanings.

#### 4.1.2 TF-IDF

TF-IDF, combining Term Frequency and Inverse Document Frequency, offers a weighted measure of word significance within documents relative to a corpus [25]. It distinguishes between common and unique terms, assigning higher weights to terms that are prevalent in a particular document but rare across the corpus. This balance between term frequency and document uniqueness aids in identifying words that are most indicative of a document’s content [26]. The TF-IDF (Term Frequency-Inverse Document Frequency) metric serves as a cornerstone in the field of text mining and information retrieval, offering a nuanced measure of a word’s relevance within a specific document relative to a collection of documents. This metric is encapsulated as shown in Eq (1).

$$\begin{array}{c} \text{TF}-\text{IDF}(t,d,D)=\text{TF}(t,d)\times \text{IDF}(t,D) \end{array}$$ (1)

where *t* denotes the term whose relevance is being evaluated, *d* represents the document containing the term, and *D* signifies the entire document corpus.

Term Frequency (TF), which quantifies the frequency of term *t* within document *d*, normalized by the total number of terms in *d*, indicating the term’s significance in the document’s context. Inverse Document Frequency (IDF), which gauges the rarity of term *t* across the corpus *D*, thereby amplifying the weight of terms that are unique to a subset of documents.

### 4.2 Dimensionality reduction and feature extraction

In addressing the complexities of managing and analyzing equipment failure data, our methodology incorporates the generation of a final embedding vector to encapsulate fault characteristics through text embeddings and associated weights. This vector is crucial for classifying text data and identifying distinct fault types. However, we encounter a significant challenge due to the high dimensionality of these embedding vectors [27]. High-dimensional datasets can complicate the analysis process in several ways: they tend to increase memory demands, prolong model training times, and add layers of complexity to the model architecture. Moreover, as the feature space expands, the distance between data points also increases, potentially triggering the so-called “curse of dimensionality.” This phenomenon can significantly impair model performance, particularly in scenarios with sparse samples, and hinders the effective use of clustering algorithms [28].

To mitigate these issues, our approach involves applying dimensionality reduction and feature extraction techniques to the final embedding vector. The objective of dimensionality reduction in this context is not merely to reduce the number of features but to do so in a manner that retains the essence of the original dataset. By preserving the core characteristics of the data, we ensure that the critical information necessary for fault classification and analysis is not lost. This process enables us to streamline the dataset, reducing its complexity while maintaining the integrity of the information it conveys. Through this refined dataset, we can more effectively apply clustering algorithms, thereby enhancing our ability to categorize faults accurately and efficiently. This strategic reduction of dimensionality is pivotal for overcoming the inherent challenges of high-dimensional text data, facilitating a more nuanced and effective analysis of equipment failures.

#### 4.2.1 SVD and truncated SVD

Singular Value Decomposition (SVD) is a sophisticated mathematical method used for decomposing a complex, high-dimensional matrix into simpler, lower-dimensional components. Specifically, this decomposition applies to any matrix *A* of size *m* × *n*, assuming *A* is not singular, facilitating its breakdown into three distinct matrices: *A* = *UΣV*^*T*^. In this representation, *A* denotes the original matrix, while *U* and *V* are orthogonal matrices, with *U* derived from the eigenvectors of *AA*^*T*^ and *V* from the eigenvectors of *A*^*T*^*A*. The matrix *Σ* is a diagonal matrix containing singular values, which are essential for understanding the structure of *A*.

The columns of *U* and *V* are known as the left and right singular vectors of *A*, respectively. These vectors are crucial for mapping the multidimensional aspects of *A* into a reduced-dimensional space, offering insights into its inherent structure.

Truncated Singular Value Decomposition (Truncated SVD) presents a more focused version of SVD by concentrating on the most significant singular values. This approach selects only the largest *K* singular values—and their corresponding vectors—to effectively reduce the data’s dimensionality to *K* dimensions. The choice of *K* is a critical parameter determined by the researcher, aiming to strike a balance between reducing complexity and preserving the original data’s key characteristics. This technique is particularly beneficial for sparse datasets as it reduces computational requirements while ensuring the core information is retained. Through Truncated SVD, a balance is achieved between simplification and the preservation of essential data features, thereby enhancing our capability to interpret and analyze high-dimensional datasets efficiently.

#### 4.2.2 AE, CAE, and VAE

Auto Encoder is an unsupervised learning algorithm that consists of an encoder and a decoder, usually a neural network. The Encoder is denoted by *Z* = *F*(*X*) and maps the input data *X* to a low-dimensional latent vector *Z*. The Decoder is denoted by *X*′ = *G*(*Z*) and uses the latent vector *Z* to generate a reconstruction of the input data *X* [29]. The latent vector *Z* generated by an AutoEncoder is not predefined, but is automatically learned as the encoder model extracts and compresses features from the data. By utilizing AutoEncoder, we can effectively reduce the dimensionality of the original data, extract the key features, and transform the data into a lower dimensional representation.

Expanding upon the Auto Encoder model, the Contractive Auto Encoder (CAE) specializes in maintaining the integrity of input data characteristics under variations and disturbances [30]. It employs a specialized loss function incorporating a penalty term, proportional to the Frobenius norm of the encoder’s Jacobian matrix at each data point, to ensure the stability of extracted features, as shown in Eq (2)

$$\begin{array}{c} L_{\text{CAE}}\left(\theta \right)=\sum_{x\in D_{n}}(L\left(x,g\left(f\left(x\right)\right)\right)+\lambda {∥J_{f}\left(x\right)∥}^{2}) \end{array}$$ (2)

The Variational Auto Encoder (VAE) represents a further evolution in the domain of autoencoders, introducing a probabilistic generative model that approximates the distribution of input data [31]. Unlike traditional autoencoders, the VAE treats the latent vector *Z* as a stochastic variable, drawing samples from a specified distribution to generate diverse representations. This model employs a regularization term in its loss function to ensure the mean and variance of the latent variables align with a predefined distribution, typically a normal distribution. This regularization facilitates the learning of a consistent distribution in the latent space, enhancing the model’s ability to generate new, yet coherent, data samples from the learned distribution space.

These models—Auto Encoder, Contractive Auto Encoder, and Variational Auto Encoder—serve as foundational elements in our study, enabling sophisticated dimensionality reduction and feature extraction. They transform complex data into more manageable forms, providing a robust framework for analyzing equipment failure data and extracting actionable insights.

### 4.3 Clustering

In this research, we delve into the utilization of unsupervised learning techniques, specifically clustering, to categorize potential failure risks within equipment maintenance data. The goal is to harness the processed and dimensionally reduced data, as discussed in prior sections, to segregate historical maintenance records into distinct risk categories. This clustering aims to enable predictive insights regarding future equipment failures, assisting managerial decision-making by providing a structured framework for risk assessment.

Clustering involves evaluating the similarities among data points and grouping them into clusters that reflect these similarities. Effective clustering results in high cohesion within clusters and distinct separation between different clusters. The choice of clustering algorithm plays a pivotal role in the analysis. Among the various algorithms available, such as K-means clustering, hierarchical clustering, density-based clustering [32], combinatorial clustering [33], K-means was selected for its computational efficiency and its suitability for our dataset.

#### 4.3.1 K-means clustering

In this study, we employed unsupervised learning, specifically K-means clustering, to classify failure risk levels due to the absence of pre-existing labels in the original dataset. K-means clustering is particularly effective for our study’s objective of categorizing failure risks. To address the FMEA problem, K-means clustering can be used to evaluate and cluster the risk of failure modes [34]. This algorithm iteratively assigns data points to clusters based on their proximity to the cluster’s centroid, ensuring a straightforward implementation and reliable convergence. In essence, K-means is a hard clustering algorithm that assigns each data point to the nearest cluster without considering the probability of membership in other clusters.

The initial step involves determining the number of clusters, *K*, which directly correlates with the identified risk levels in our study. The selection of *K* is critical and must be approached with a method that balances the need for detailed risk differentiation with the practicalities of dataset size and diversity. Also, centroids serve as the central points of each cluster, and since performance can fluctuate significantly depending on their selection, the process of initializing centroids is crucial. The challenge of determining suitable initial centroids has been recognized as a common limitation in K-means clustering, and recent research has proposed various approaches to address this, [35–37] to enhance clustering performance. In this study, we employed random initialization; however future research may explore the application of various centroid initialization methods to systematically analyze performance in domain-specific risk assessments. Such an experimental approach is expected to address the limitations of traditional initialization in K-means clustering and contribute to developing more precise risk assessment techniques tailored to the characteristics of each domain.

#### 4.3.2 Silhouette score

Determining the optimal *K* for K-means clustering involves the use of the Silhouette Score, a metric that assesses the quality of clustering by measuring how similar an object is to its own cluster compared to other clusters. The score ranges from -1 to +1, where a score close to +1 indicates a well-matched object to its own cluster and distinct separation from others. This score provides an objective criterion for selecting the most appropriate *K*, enhancing the reliability of our clustering outcomes.

Utilizing the scikit-learn library, we can easily compute the Silhouette Score for different values of *K*, enabling an informed selection of the optimal cluster count for our analysis. This methodological approach underpins our clustering analysis, ensuring that the derived risk categories are both statistically valid and practically relevant for enhancing equipment maintenance strategies.

To calculate the silhouette coefficient, for all data points *i*, the average distance between the point and other points in the same cluster is called *a*_*i*_, and the average distance to the nearest cluster, excluding the cluster to which the point belongs, is called *b*_*i*_. Then, using Eq (3), we can calculate the silhouette score of each data point present. In fact, the process of checking for good clustering for the entire data is represented by a single value in the silhouette score. This is because it is the average of the silhouette scores of all the data points using Eq (4). Therefore, we can look at this value and judge the performance of the clustering using the aforementioned interpretation method.

$$\begin{array}{c} S\left(i\right)=\frac{b_{i}-a_{i}}{\text{max}(a_{i},b_{i})} \end{array}$$ (3)

$$\begin{array}{c} \text{Silouette}\;\text{Score}=\frac{\sum_{i=1}^{n}S\left(i\right)}{n} \end{array}$$ (4)

### 4.4 Topic modeling

Following the clustering and risk assessment of various equipment failures, our study advances to employ topic modeling techniques to decipher the themes and underlying nature of these identified risks. Topic modeling serves as a powerful statistical tool for distilling potential topics from sets of unstructured documents, effectively unveiling the latent thematic structure within large text corpora.

Latent Dirichlet Allocation (LDA) [16] stands out as the predominant method in the realm of topic modeling. LDA operates under two fundamental assumptions: firstly, that each document is a mixture of various topics, and secondly, that each topic is characterized by a distribution of words. This approach employs the Bag of Words and TF-IDF methodologies, implying that LDA does not account for word order within the documents. In practice, LDA requires the pre-specification of the number of topics, denoted as *K*, as a critical hyperparameter. Upon configuration, LDA initiates by randomly associating each word in the document to a topic. It then iteratively refines these assignments through Gibbs Sampling, a process that re-evaluates the topic of each word by considering both the distribution of topics across the document and the prevalence of words within those topics, until a stable topic assignment for all words is achieved.

Despite its widespread application, LDA’s probabilistic foundation—which treats all words within a document as independent occurrences—introduces certain limitations. Specifically, LDA’s inability to capture the contextual nuances surrounding word usage and its oversight of inter-topic correlations [38] pose challenges. Given the intricate web of interactions and sequences of events characterizing equipment failure incidents, the direct application of LDA for topic modeling within this domain encounters obstacles in accurately capturing the complex, context-dependent relationships inherent in failure narratives. This underscores the need for sophisticated approaches that can navigate the subtleties of context and correlation, enhancing the precision and depth of topic modeling in failure analysis.

#### 4.4.1 BERTopic

In this study, we employ BERTopic [18], an innovative topic modeling technique designed to address the shortcomings of traditional methods like LSA and LDA. BERTopic, as the name suggests, is a topic modeling technique that leverages BERT [39] embeddings in conjunction with a class-based TF-IDF approach [18]. This method generates contextualized word and sentence vector representations using BERT, and subsequently extracts topics through a modified application of TF-iDF. A key advantage of BERT is its ability to produce representations that capture the bidirectional context between words, thus providing superior contextual understanding [40]. This characteristic makes BERTopic particularly effective in extracting nuanced and coherent topics from text data. BERTopic enhances topic modeling through a three-step process:

1. Document Embeddings: Utilizes Sentence BERT to transform documents into embedding representations, capturing deep semantic meanings.
2. Document Clustering: Applies UMAP (Uniform Manifold Approximation and Projection) for dimensionality reduction, followed by HDBSCAN for clustering, effectively grouping documents by thematic similarity.
3. Topic Representation: Constructs topics using a class-based TF-IDF, focusing on the significance of words within clusters, for precise topic delineation.

BERTopic’s methodology, integrating advanced embedding, reduction, and clustering techniques, outperforms traditional topic modeling by capturing the nuanced context of text data. This approach is particularly beneficial for our analysis of equipment failure data, allowing for a detailed exploration of risk-related topics within classified clusters. Through BERTopic, we gain deeper insights into failure mechanisms, significantly enhancing our understanding and management of equipment risks.

## 5 Data preprocessing

### 5.1 Dataset

For this study, facility maintenance records spanning from August to December 2014 and January to November 2016 were consolidated into a comprehensive dataset. This dataset encompasses information across 8 units, detailing partial failures, incident timings, downtime durations, failure causes, remedies implemented, and performance metrics. The consolidated file features columns such as Exhalation, Part, Date, Start Time, Finish Time, Cause of Failure, Remedy, MTTR (Mean Time To Repair), MTBF (Mean Time Between Failures), and Failure Intensity Rate. To facilitate risk level differentiation based on failure impact and likelihood, additional columns, namely risk_impact and risk_likelihood, were introduced, resulting in a dataset structured around 12 pivotal columns.

The dataset delineates eight distinct units and six parts: extruder, furnace, puller, rear, hot saw, and others, as shown in Table 1. MTTR and MTBF serve as critical reliability indicators, quantifying average repair durations and intervals between failures, respectively. The Fault Intensity Rate offers insight into equipment downtime due to failures, thereby aiding in equipment preservation and maintenance efficiency assessments.

**Table 1:** Dataset description.

Columns	Types	Description
Equipment Number	Number	Number of each Equipment (e.g., “Unit 1”, “Unit 7)
Part	Text	Components of the Equipment (“Extruder”, “Heater”, “Backside”, “Hot Saw”, “Puller”, “Misc.”)
Date	Date	The Date of the Failure (e.g., “28.Mar”, “11.Apr”, etc)
Start Time	Time	Time when equipment got failure (e.g., “18:00:00”, “13:45:00”, etc)
Finish Time	Time	Time when equipment got fixed (e.g., “18:40:00”, “17:17:00”, etc)
Stopped Time(min)	Number	Time interval from failure to fixed (e.g., 40, 212, etc)
Stopped Time(hour)	Number	Time interval from failure to fixed (e.g., 0.67, 3.53, etc)
Cause of Failure	Text	The main cause of failure (e.g., “Main and booster pump trip”, “Container main piping block”, etc)
Failure Handling	Text	The method of fixing failure (e.g., “Knocker cable shor circuit due to worker’s carelessness”, “Full range bolt shear seal and bolt replacement”, etc)
MTTR	Number	Mean Time To Repair (e.g., 1.16, 2.19, etc)
MTBF	Number	Mean Time Between Failure (e.g., 50.8, 82.8, etc)
Failure Strength Ratio	Number	Breakdown durability rate (e.g., 2.29, 2.65, etc)
Price weighting by part	Number	Distribution of the price and importance of the part (e.g., 0.35,0.25, etc)
Risk Impact	Number	Metrices that mesures the intensity of failure (e.g, 4.4812, 4.8417, etc)
Risk Likelihood	Number	Metrices that mesures how often equipment fails (e.g., 3.9982, 3.0004, etc)

### 5.2 Weighting by part

Aluminum extrusion requires a wide variety of equipment and devices, and the importance and value of each can vary. In this study, the price weighting of major equipment used in aluminum extrusion companies is examined in depth. The price of equipment serves as a crucial indicator that comprehensively reflects the technical complexity, energy consumption, and maintenance requirements of the equipment. This signifies that equipment plays a role beyond a mere physical machine, and as more advanced technologies are employed, the importance of the functions that the equipment performs increases. For instance, in the aluminum extrusion process, an extruder directly influences product quality and productivity while heater plays a decisive role in controlling the overall efficiency of the process through temperature regulation during extrusion. Hence, equipment prices are set at a high level, as these pieces of equipment fulfill a critical role in the process.

Optimizing equipment maintenance and energy efficiency is a vital factor in process operations [41]. This implies that equipment prices serve as a significant indicator not only of initial purchase costs but also of long-term maintenance and operational expenses. While performance or maintenance frequency could also be used as weighting factors, these metrics may vary over time or depending on the process environment. Therefore, using price as a weighting factor was deemed the most appropriate method for evaluating the overall operating costs and importance of the equipment.

First, we investigated the composition of equipment prices based on internal company information and data. We found that extruders and furnaces together account for about 60% of the total equipment price. This is due to the pivotal role that extruders and furnaces play in the extrusion process. There were also differences in prices for the backside, hot saw, puller, and other parts. In particular, we believe that the functionality of each part and its role in the production process may have contributed to these price differences. Based on the above results, we propose a weight distribution based on 100 for setting price weights. Specifically, we concluded that the extruder should be weighted 35, the heater 25, the backside 15, the hot saw 10, the puller 9, and the other parts 6, as shown in Table 2. This distribution reflects the importance and functionality of the equipment.

**Table 2:** Price weighting by part.

Part	Extruder	Heater	Backside	Hot Saw	Puller	Misc.
**Weight**	35	25	15	10	9	6

### 5.3 Risk impact conversion function

Within the industrial sector, the operational integrity of equipment plays a pivotal role in determining the efficiency and competitiveness of a company. The seamless functioning of machinery is intrinsically linked to critical operational outcomes, including the volume of production, the assurance of product quality, and the precision of delivery schedules. Consequently, any instance of equipment failure emerges as a significant threat, undermining these essential variables and, by extension, potentially diminishing production efficiency and eroding the competitive edge of the business.

The repercussions of equipment malfunctions extend well beyond the immediate disruption of production processes. The aftermath of a breakdown necessitates repair or maintenance interventions, incurring additional expenditures for part replacements, lubricant purchases, and other related costs. Moreover, the occurrence of equipment failures elevates the risk to workplace safety, posing potential hazards that could lead to work stoppages, endanger worker safety, or result in material damage.

In response to these operational challenges, the industry has developed and employed various metrics and methodologies aimed at quantifying and mitigating the impact of equipment failures. Among these, the Mean Time to Repair (MTTR) stands out as a critical metric, offering insight into the repair and recovery time of equipment relative to its operational uptime. By applying a weighted analysis based on the MTTR as shown in Eq (5), a nuanced assessment of the risk impact associated with equipment failures can be achieved, facilitating more informed decision-making processes.

Further enhancing this analytical framework, the Maximum Time To Repair (MaxTTR) as shown in Eq 6 and the application of Gaussian distribution models as shown in Eq 7 are utilized to evaluate the repair risk rate or Forecast Error as shown in Eq 8. The Gaussian distribution, in particular, serves as a predictive tool, enabling the estimation of risk rates by analyzing historical repair patterns and failure frequencies. This predictive capability is instrumental in guiding the development of strategic plans for facility management and maintenance, ensuring that proactive measures are in place to address potential equipment failures and minimize their impact on production continuity and workplace safety.

$$\begin{array}{c} \text{MTTR}=\frac{\text{Total}\;\text{repair}\;\text{time}}{\text{Total}\;\text{number}\;\text{of}\;\text{failures}} \end{array}$$ (5)

$$\begin{array}{c} \text{MaxTTR}=\text{MTTR}(1+1.96\times \sigma_{p}) \end{array}$$ (6)

$$\begin{array}{c} \sigma_{p}^{2}=\frac{1}{N}{\bar{\sigma }}^{2}+\frac{N-1}{N}\bar{\text{COV}}\left(R_{i},R_{j}\right) \end{array}$$ (7)

$$\begin{array}{c} FE=\sqrt[{}]{\sigma_{l}^{2}+\sigma_{p}^{2}} \end{array}$$ (8)

### 5.4 Risk likelihood conversion function

In the realm of industrial operations, the frequency of equipment failures within a given year stands as a critical indicator of a facility’s operational integrity. This metric serves as a cornerstone for evaluating a facility’s reliability and for crafting maintenance strategies that bolster operational efficiency. The total operational uptime of a facility plays a crucial role in this assessment, offering a backdrop against which the frequency of failures is evaluated.

Furthermore, an essential metric for a more nuanced analysis of equipment failures is the Mean Time Between Failures (MTBF) as shown in Eq 9. MTBF is extensively applied in gauging the reliability of equipment, providing an average duration of operational reliability across an extended timeframe. This measure is instrumental in delivering an objective assessment of equipment quality and performance capabilities as shown in Eq 10.

$$\begin{array}{c} \text{MTBF}=\frac{\text{Total}\;\text{operational}\;\text{time}}{\text{Number}\;\text{of}\;\text{failures}} \end{array}$$ (9)

$$\begin{array}{c} \text{Number}\;\text{of}\;\text{failures}\;\text{per}\;\text{year}=\frac{\text{Total}\;\text{uptime}}{\text{MTBF}} \end{array}$$ (10)

### 5.5 Text pre-processing

Textual data from the dataset underwent rigorous preprocessing, including translation from Korean to English, normalization, and keyword extraction. Utilizing NLP techniques, the study processed failure causes, remedies, and other textual data to prepare them for analysis, employing text embedding to convert this information into a machine-readable format. The Python’s googletrans library facilitated the translation of textual data, ensuring accessibility and uniformity across the dataset. Following translation, text data was standardized and refined through steps such as lowercasing, tokenization, and stopword removal, culminating in a clean, analyzable format conducive to subsequent embedding and analysis.

### 5.6 Generating input data

To develop a model that determines the location of new failures and the expected failure grade based on the failure content, each location was translated into English, and the failure cause column was combined to create an input column. The final input data was generated by assigning new weights to the sentence vectors generated by using Word2Vec and TF-IDF algorithms for the input columns. The weight values were obtained by converting risk impact and risk likelihood. Finally, the final embedding vector containing the impact and frequency rate of each failure on the facility was generated.

## 6 Experiments

### 6.1 Dimensionality reduction and feature extraction

The final embedding vector represents the characteristics of each fault based on the embeddings and weights of the text. However, the high dimensionality of the final embedding vector is one of the challenges in text data classification [31]. When the dimensionality of the features representing the characteristics of the data is large, various problems can arise. First, high-dimensional data can increase memory usage, slow down training, and increase the complexity of the model. Additionally, as the dimensionality increases, the distance between the data increases, which can lead to a dimensionality curse that degrades the performance of the model, especially when samples are sparse. This curse of dimensionality prevents the meaningful application of clustering algorithms [42].

Therefore, we tried to solve the above problems by performing dimensionality reduction and feature extraction of the final embedding vector. In dimensionality reduction, we do not simply reduce the dimensionality of the data, but we try to preserve the characteristics of the original data as much as possible, so we can extract the main characteristics of the data. We applied four models to the data as dimensionality reduction methods: Truncated SVD, AutoEncoder, Convolutional AutoEncoder (CAE), and Variational AutoEncoder (VAE), and compared five outputs, including the original data without dimensionality reduction.

### 6.2 Setting truncated SVD hyperparameters

The hyper-parameter K, which should be set to reduce dimensionality and extract features based on Truncated SVD, was chosen based on the Explained Variance Ratio as shown in Table 3. The Explained Variance Ratio is one of the outliers divided by the sum of all the outliers, the larger the value, the more the component explains the total variance. The explained variance ratio for each dimension indicates the proportion of the total variance that dimension accounts for, and the cumulative explained variance ratio is the cumulative value for each dimension.

Therefore, the hyperparameter K was set to values of 0.75, 0.8, 0.85, 0.9, and 0.95, so that the cumulative explained variance ratio could account for 75%, 80%, 85%, 90%, and 95% of the total data variance, respectively. The hyperparameter K with the highest silhouette score was then selected based on the experiments. Consequently, the final embedding using Word2Vec had a cumulative explained variance ratio of 75%, reducing the original 100 dimensions to 10 dimensions. Similarly, the final embedding using TF-IDF had a cumulative explained variance ratio of 80%, reducing the original 622 dimensions to 107 dimensions. This allowed us to effectively reduce the data while preserving important features.

**Table 3:** Hyperparameter of explained variance ratio.

Feature Extractor	Variance Ratio(K)	Reduced Dimension	Best Silhouette Score	Number of Cluster
Word2Vec	75	10	0.415	3
Word2Vec	80	15	0.39	3
Word2Vec	85	23	0.367	3
Word2Vec	90	33	0.348	3
Word2Vec	95	51	0.33	3
TF-IDF	75	89	0.384	5
TF-IDF	80	107	0.385	4
TF-IDF	85	131	0.319	4
TF-IDF	90	168	0.326	5
TF-IDF	95	230	0.299	6

### 6.3 Failure risk ordering by clustering

After clustering by failure cause, we need a quantitative indicator of risk per cluster. For this metric, we used the average of the cluster-specific failure intensity rates per failure in the raw data. Fault intensity rate is the percentage of time a facility is down due to a fault, expressed as the sum of fault downtime / total uptime. Cycles are typically expressed in months per unit of time.

**Fig 6:**
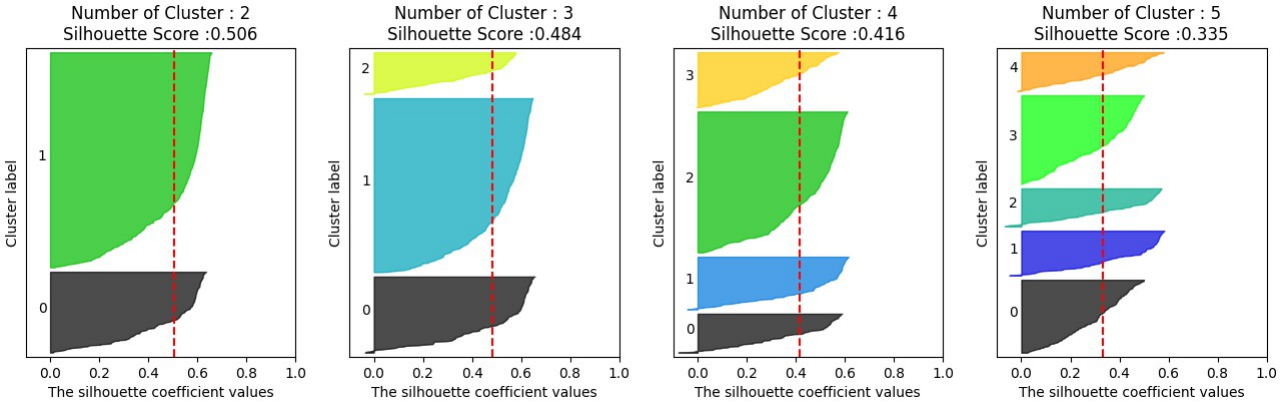
Visualize Silhouette with Word2Vec.

**Fig 7:**
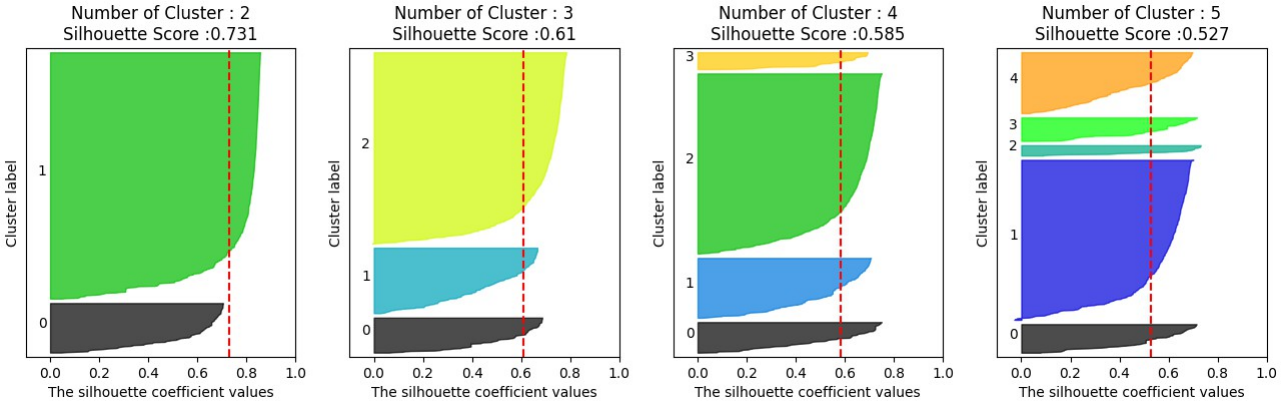
Visualize Silhouette with TF-IDF.

Word2Vec and TF-IDF methods were used for word embedding, and None, Truncated SVD, AutoEncoder, Contractive AutoEncoder, and Variational AutoEncoder for feature extraction, and Silhouette Scores from cluster size 3 to 9 were compared for each. We decided that it was not meaningful to have two risk levels, so we only visualized the Silhouette Score and excluded the comparison group. In total, we compared 70 Silhouette Scores and chose the method with the highest value. In our analysis, Fig 6 presents a visualization that highlights the optimal Silhouette Scores achieved through the application of the Word2Vec technique. Similarly, Fig 7 showcases a visualization of the top Silhouette Scores obtained by employing the TF-IDF method.

**Fig 8:**
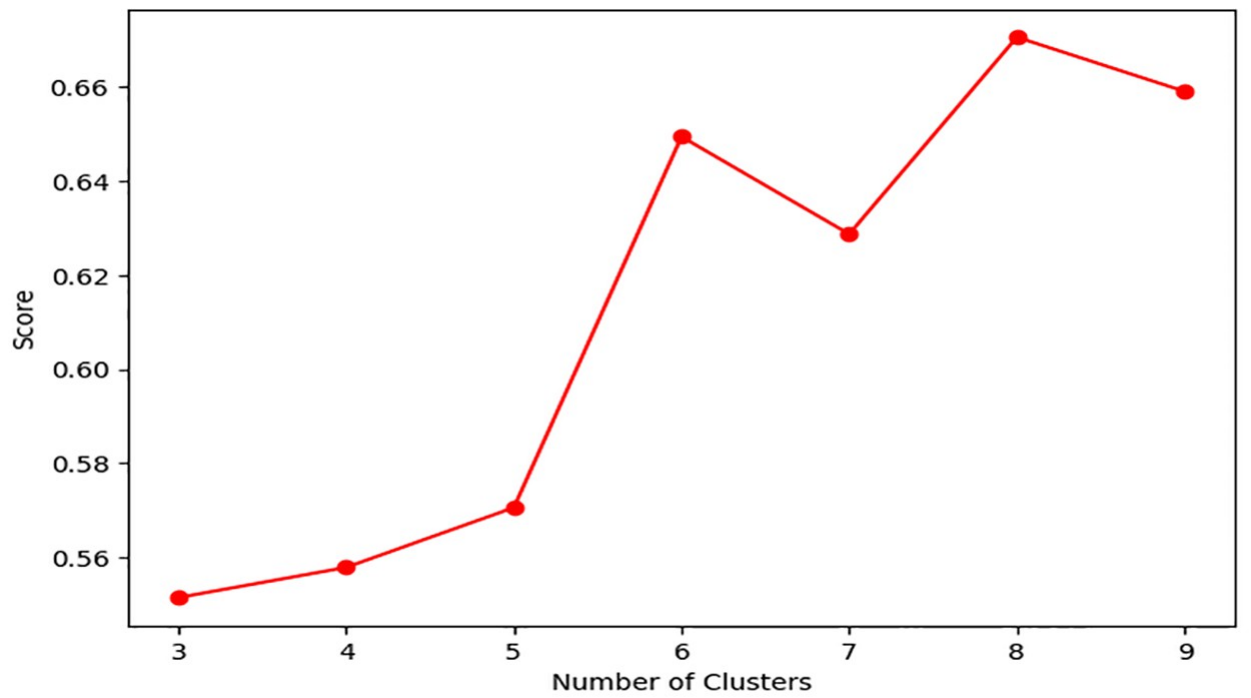
Davies-Bouldin index.

**Fig 9:**
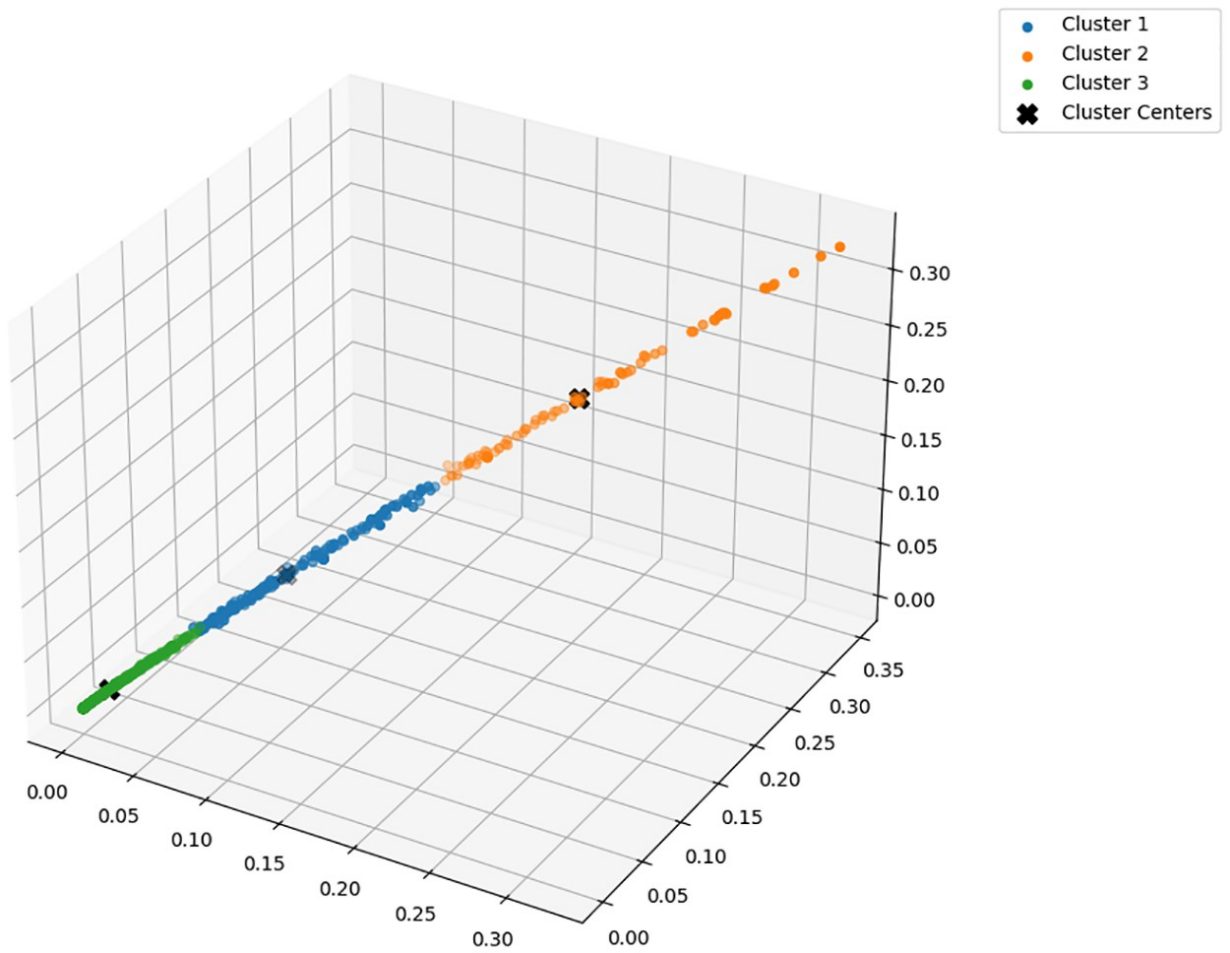
3D visualization results of risk-based clustering.

**Fig 10:**
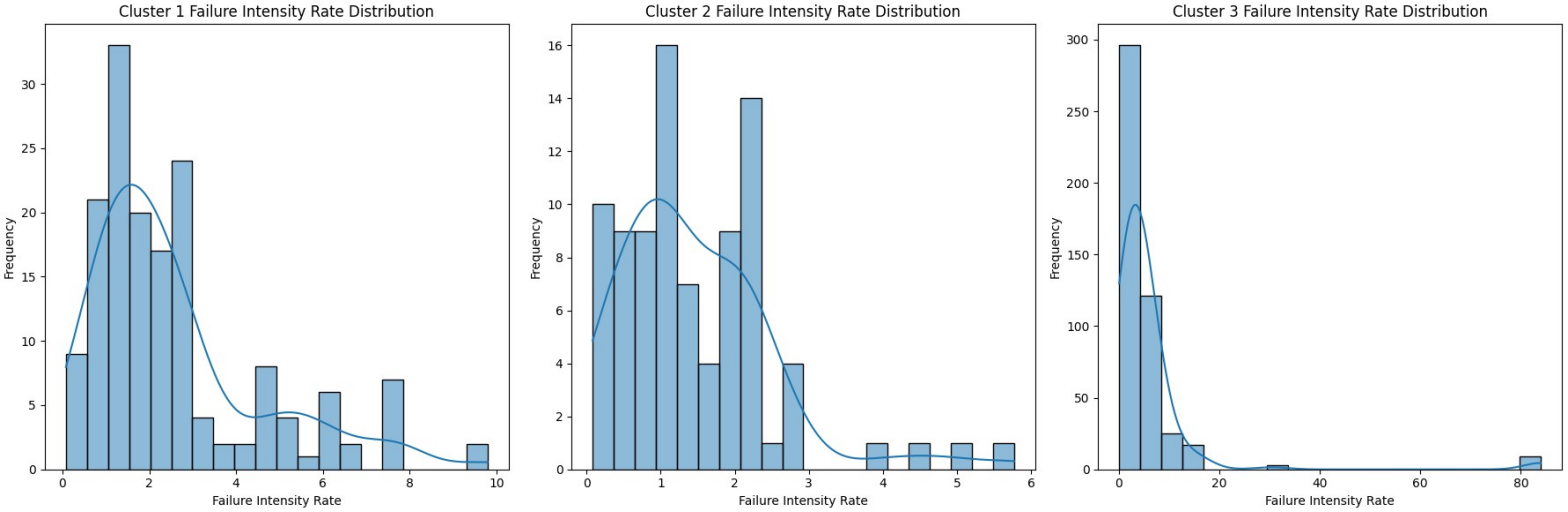
Failure strength rate distribution.

The highest Silhouette Score of 0.61 was obtained with Word2Vec for Word Embedding, Contractive AutoEncoder for Feature Extraction, and 3 clusters. To further validate the selection of 3 clusters as the optimal number, we additionally evaluated the model using the Davies-Bouldin Index, which measures cluster compactness and separation. And smaller the value of the Davies-Bouldin calculation results, the more accurate the K value is used [43]. The Davis-Bouldin Index for 3 clusters was found to be the lowest, as shown in Fig 8, confirming that both the Silhohouette score and the Davies-Bouldin index support the selection of 3 clusters as the optimal configuration. The clustering results are shown in Table 4. A three-dimensional visualization of each document in this way as a scatter plot is shown in Fig 9. A chart visualizing the distribution of failure intensity rates for each cluster is shown in Fig 10.

**Table 4:** Clustering results.

Cluster Number	Average Failure Strength Ratio	SD	Number	Risk Level
Cluster 1	2.59	2.01	162	2
Cluster 2	1.46	1.04	87	1
Cluster 3	5.84	11.52	471	3

The lower the average failure intensity rate, the more reliable the equipment is, and the more efficient the maintenance is. maintenance efficiency. The more dangerous failure clusters have higher risk levels, which means that cluster 2 has the highest risk level of 3, cluster 3 has the next highest risk level of 2, and so on. Cluster 2 has the highest risk level of 3, Cluster 3 has the next highest risk level of 2, and Cluster 1 has the lowest risk level of 1. 1, the lowest risk level.

To evaluate whether there is a statistically significant difference between the categorized clusters, we first conducted the Shapiro-Wilk test to assess the normality of each cluster. Using the shapiro function from the scipy.stats module in Python, we calculated the p-values for each of the three clusters as 4.3 × *e*^−12^, 6.91 × *e*^−7^, and 2.31 × *e*^−38^, respectively. All p-values were below the predefined significance level of 0.05, thereby rejecting the null hypothesis that the data follows a normal distribution. This result indicates that the data in each cluster does not satisfy the assumption of normality.

Subsequently, we employed the Kruskal-Wallis H test, a non-parametric method, to determine if there are differences in the medians across the three or more groups. The kruskal function from the scipy.stats module was used, with a significance level of 0.05. The test yielded a Kruskal-Wallis statistic of 147.18 and a p-value of 1.09 × *e*^−32^, which is below the significance threshold. This result allows us to reject the null hypothesis that the medians of all clusters are equal, suggesting that there are significant differences among the cluster medians.

For post hoc analysis, we conducted Pairwise Wilcoxon Rank-Sum Tests to identify specific differences in medians between each cluster pair. To address the issue of multiple comparisons, we applied the Bonferroni correction using the multipletests function from the statsmodels.stats.multitest module in Python. THe adjusted p-values for the comparisons between Cluster 1 and Cluster 2, Cluster 1 and Cluster 3, and Cluster 2 and Cluster 3 were 2.07 × *e*^−6^, 1.19 × *e*^−13^, 3.67 × *e*^−26^, respectively.

Furthermore, we performed additional pairwise comparisons using the mannwhitneyu function from the scipy.stats module, which yielded p-values of 6.91 × *e*^−7^, 3.95 × *e*^−14^, and 1.22 × *e*^−26^ for the comparisons between Cluster 1 and Cluster 2, Cluster 1 and Cluster 3, and Cluster 2 and Cluster 3, respectively. All these p-values were below the adjusted significance level, confirming that the median differences between all cluster pairs are statistically significant. This suggests that the fault severity ratios differ significantly among the cluster, and that the clusters have been appropriately categorized according to their risk levels.

### 6.4 BERTopic visualization and analysis by risk

#### 6.4.1 Risk level 1 (Cluster 2)

**Fig 11:**
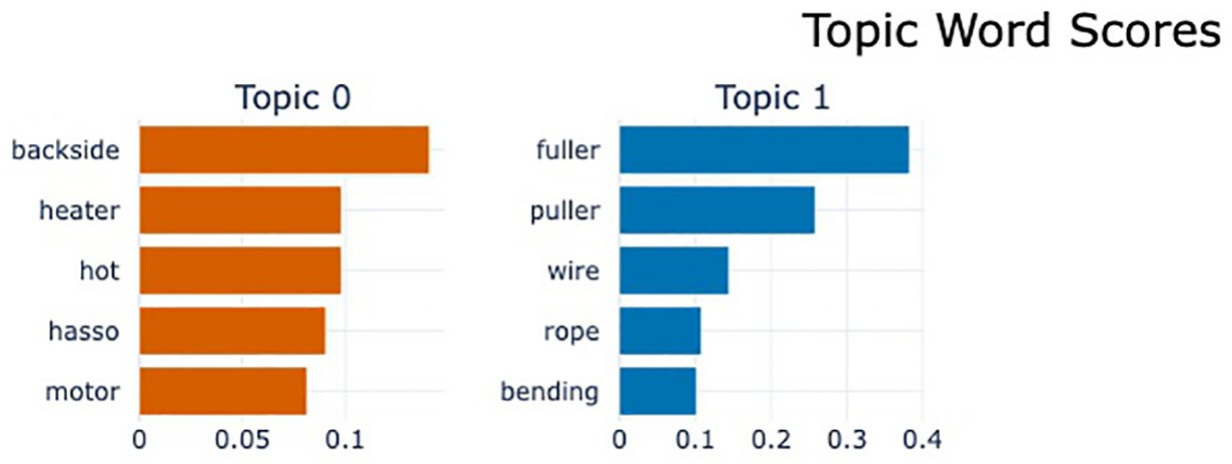
Word scores by topic for risk level 1.

For Cluster 2, the sample size is too small to properly visualize the topics, as shown in Fig 11. However, since this is the cluster with the lowest risk, and failures with low risk are less important to analyze, we analyzed Cluster 1 and Cluster 3 with higher risk and a larger number of topics.

#### 6.4.2 Risk level 2 (Cluster 1)

**Fig 12:**
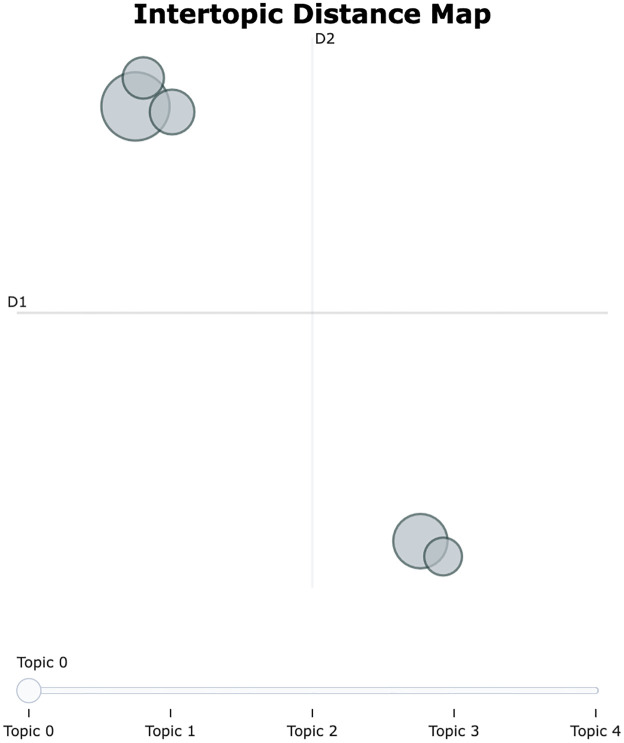
Intertopic distance map for risk level 2.

**Fig 13:**
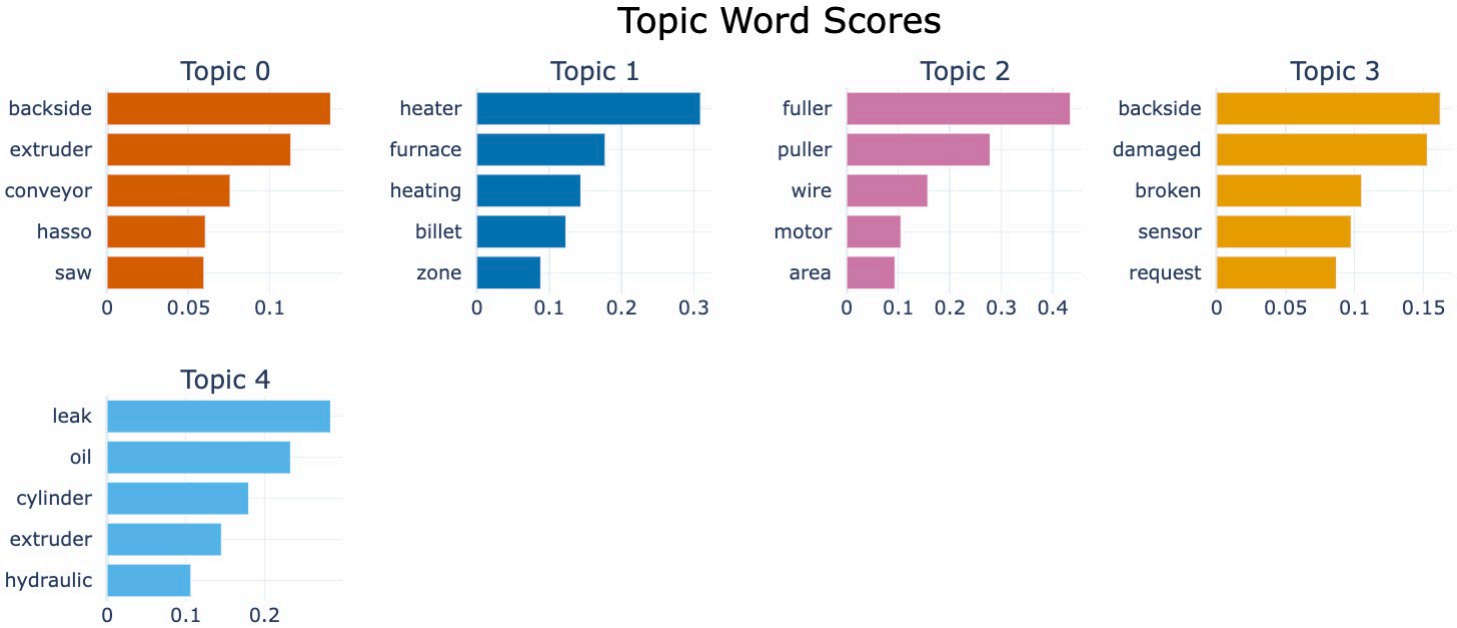
Word scores by topic for risk level 2.

Based on the analysis results, a total of five topics were identified, and strategic directions were sought to improve the efficiency and quality of the extrusion process through their interrelationships. The topic visualization shows that Topics 0,2,3 are adjacent and Topics 1,4 are adjacent, as shown in Fig 12. Fig 13 shows the importance of words in each topic.

First, it was confirmed that Topics 0, 2, and 3 are closely related. These topics are centered around key equipment and components used within the extruder. For example, the terms backside, extruder, and conveyor that appeared in Topic 0 play a critical role in the movement and quality inspection of extruded products. In particular, the backside is responsible for inspecting the thickness and shape of the extruded product, and if the performance of this equipment deteriorates, it can have a severe impact on product quality. In such situations, components like fuller, puller, wire, and motor in Topic 2 regulate the flow and movement of materials inside the extruder, significantly affecting the efficiency of the extrusion process.

Topic 3 addresses issues related to equipment damage and breakdowns (damaged, broken), which are directly associated with the performance degradation of equipment like the backside. Additionally, the inclusion of terms such as sensor and request suggests the need for monitoring equipment performance and making maintenance requests. In other words, these topics explain how problems that arise from the interactions between equipment in the extrusion process can lead to a decrease in product quality.

Meanwhile, Topics 1 and 4 primarily consist of equipment related to material heating and hydraulic systems, and their interrelationship is also clearly depicted. In Topic 1, heater and furnace are key devices used for heating materials in the extruder, and if these devices do not operate properly, the quality of the extruded product can deteriorate. During the heating process, the conditions of the billet and zone also play a crucial role; if these elements are in poor condition, the material may be heated unevenly, adversely affecting the product quality.

Topic 4 addresses issues related to hydraulic systems, including key components such as the cylinder and hydraulic, as well as oil leakage problems. The hydraulic system issues highlighted in this topic can be a major cause of performance degradation in the extruder, and oil leaks, in particular, can lower system pressure, reducing the efficiency of the equipment. If the hydraulic system does not function properly, components such as the cylinder are likely to be damaged, which can lead to a decline in the extruder’s overall performance. Conversely, a decrease in cylinder performance can also negatively impact the hydraulic system.

Overall, it can be observed that the various issues arising in the extrusion process are interconnected through the close interactions between equipment and components. Specifically, the equipment that manages material flow and quality inside the extruder operates in conjunction with each other, and if the performance of even one of these is compromised, it can significantly impact the efficiency of the entire process. Additionally, the relationship between material heating and the hydraulic system also plays a crucial role in process stability and quality management. Therefore, to optimize the extrusion process, it is essential to adopt a maintenance strategy and a systematic approach that considers these equipment interactions.

#### 6.4.3 Risk level 3 (Cluster 3)

**Fig 14:**
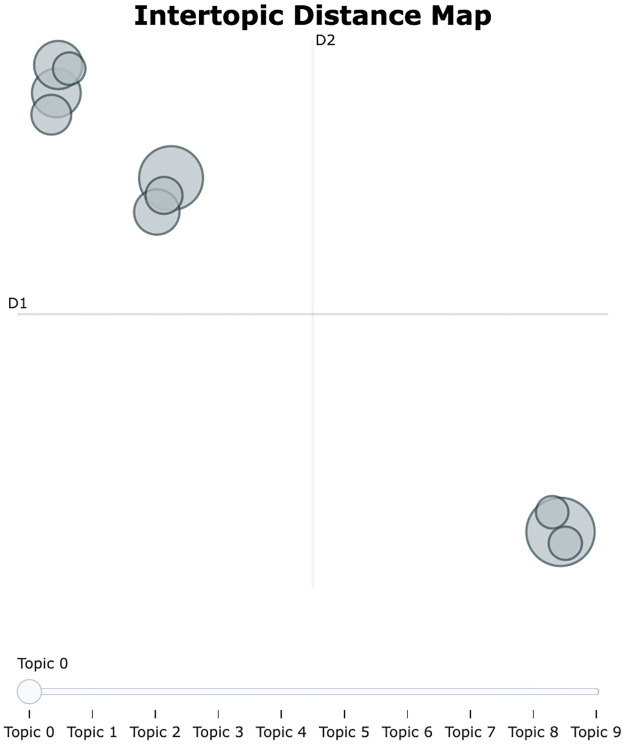
Intertopic distance map for risk level 3.

**Fig 15:**
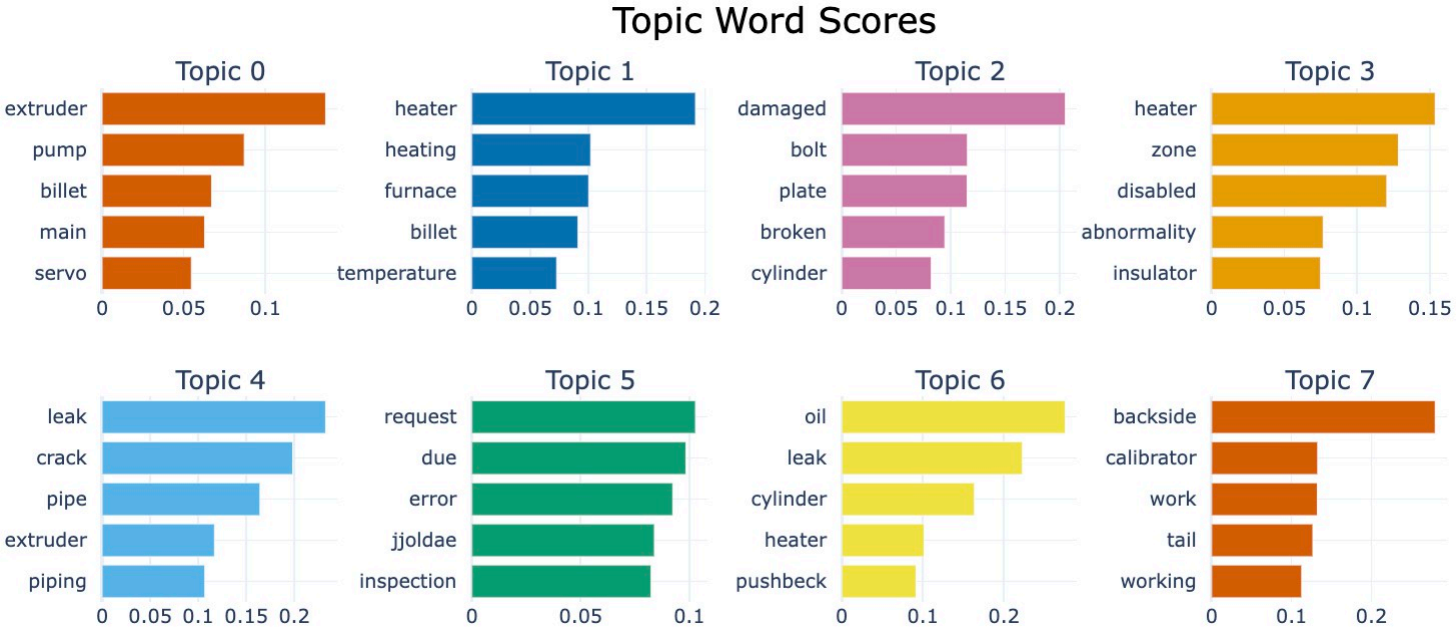
Word scores by topic for risk level 3.

Through visualization, a total of 8 key topics were derived, along with an additional analysis of Topic 8 and Topic 9. Given the domain and volume of data, the number of topics generated by BERTopic can be extensive. Therefore, the analysis primarily focuses on the top 8 topics, with additional topics considered for reference. This analysis explored the interactions throughout the extrusion process, covering equipment damage, maintenance requests, and product quality management. The topic visualization shows that Topics 2, 3, 5 are adjacent, Topics 1, 4, 6 are adjacent, and Topics 0, 7 are adjacent, as shown in Fig 14. Fig 15 shows the importance of words by topic for risk level 3. Additionally, Topic 8 is grouped with Topics 2, 3, and 5, and Topic 9 is associated with Topics 0 and 7.

First, Topic 2, Topic 3, and Topic 5 clearly illustrate the relationship between equipment damage and maintenance requests. Abnormal conditions in heaters and temperature control devices are directly linked to a decline in equipment performance, necessitating maintenance. For instance, abnormal states occurring in the heater or zone can lead to failures in temperature control, which can manifest as damage to components like bolts or cylinders. Such damaged parts eventually require maintenance requests and regular inspections, which are essential for maintaining process stability.

On the other hand, Topic 1, Topic 4, and Topic 6 emphasize the interaction between material heating and the hydraulic system. If the performance of the material heating device deteriorates, it can affect the quality of the product, and issues such as leaks or cracks can hinder proper material flow within the extruder. Leaks in the hydraulic system can impact the performance of the cylinder and heater, negatively affecting the overall performance of the extruder. Monitoring equipment performance continuously and preventing issues in advance through this interaction is crucial. Problems in the hydraulic system can lower process pressure, leading to a decline in product quality, and leaked oil can be a cause of equipment damage.

Topic 0 and Topic 7 demonstrate the correlation between major components in the extruder and product quality management. The extruder, pump, and servo must function smoothly within the process, as any performance degradation in these components directly affects product quality. During the correction and adjustment processes, equipment like the backside or calibrator plays a significant role in measuring the thickness and shape of the product, and managing the final quality. Relatedly, Topic 9 addresses various factors occurring during the product quality measurement process, including issues such as operational changes during the process. Terms like gauge and operation suggest that different tasks in the product quality inspection process are interacting with each other.

Additionally, the analysis of Topic 8(’cannot’,’move’,’forward’,’ram’,’backward’) and Topic 9(’possible’,’operation’,’gauge’,’head’,’reverse’) shows interactions similar to those in Topic 2, 3, 5 and Topic 0, 7, respectively. With the inclusion of Topic 8, it becomes evident that issues such as the inability of equipment to move or the Ram failing to move forward and backward are directly related to damaged parts or faulty equipment, exemplifying the potential problems arising from interactions between equipment in the extrusion process. Topic 9 addresses factors occurring in the product quality management process, clearly demonstrating how major equipment in the extruder operates and how this ultimately determines the final product quality.

In conclusion, interactions between equipment and components play a critical role in the quality and stability of the extrusion process. Damaged equipment or components trigger maintenance requests, and issues in the material heating devices or hydraulic systems have a direct impact on product quality. Therefore, it is essential to identify these interactions in advance and ensure process stability through proper maintenance. This approach will help optimize the process and maintain the performance of equipment and components at an optimal level.

### 6.5 Evaluation of research applicability by industry experts

Additionally, to evaluate the industrial applicability of the proposed method, qualitative validation was conducted using a survey with 10 experts, including practitioners from the manufacturing field and professors in the fields of industrial and mechanical engineering. The survey used for validation consisted of 10 items, which were categorized into three main areas: the usefulness of the research results, the effectiveness of fault diagnosis and prevention, and the applicability of the method in real-world settings. Each item was rated on a 5-point Likert scale, where 1 indicated “strongly disagree” (negative) and 5 indicated “strongly agree” (positive), allowing respondents to express either positive or negative opinions about the proposed method. The survey results were analyzed using the Wilcoxon Signed-Rank Test, a non-parametric statistical method appropriate for small sample sizes. The test was applied to determine whether the responses for each item were neutral or exhibited a statistically significant positive/negative bias. In this study, the distribution of responses for each item was evaluated against a neutral response (3 on the Likert scale) to assess whether the expert and practitioner responses indicated neutral tendencies or positive/negative trends. The survey questions are as follows:

- Utilization of Research Findings
  - Q2: The fault risk classification based on the research results can be effectively utilized in equipment management.
  - Q4: The research outcomes assist facility managers in anticipating and responding to potential faults in advance.
  - Q6: The analyzed results from the study can be useful for analyzing the causes of equipment failures and implementing preventive measures.
  - Q9: The findings of the research contribute to improving the efficiency of maintenance tasks in manufacturing facilities.
- Superiority of Fault Diagnosis and Prevention
  - Q3: The fault risk classification derived from the research results enables the clear distinction of different fault types.
  - Q8: The proposed method offers better results compared to the currently used fault diagnosis and management methods.
- Applicability in Industry
  - Q1: The method proposed in the study appears to be effective in solving machine failure issues encountered in real-world scenarios.
  - Q5: The method of categorizing fault risk is deemed appropriate for application in actual facility management tasks.
  - Q7: The fault diagnosis and prevention system improvements based on the research findings are expected to work effectively in practical settings.
  - Q10: The approach suggested in the research is considered practical enough to be adopted and implemented in real-world industrial environments.

**Fig 16:**
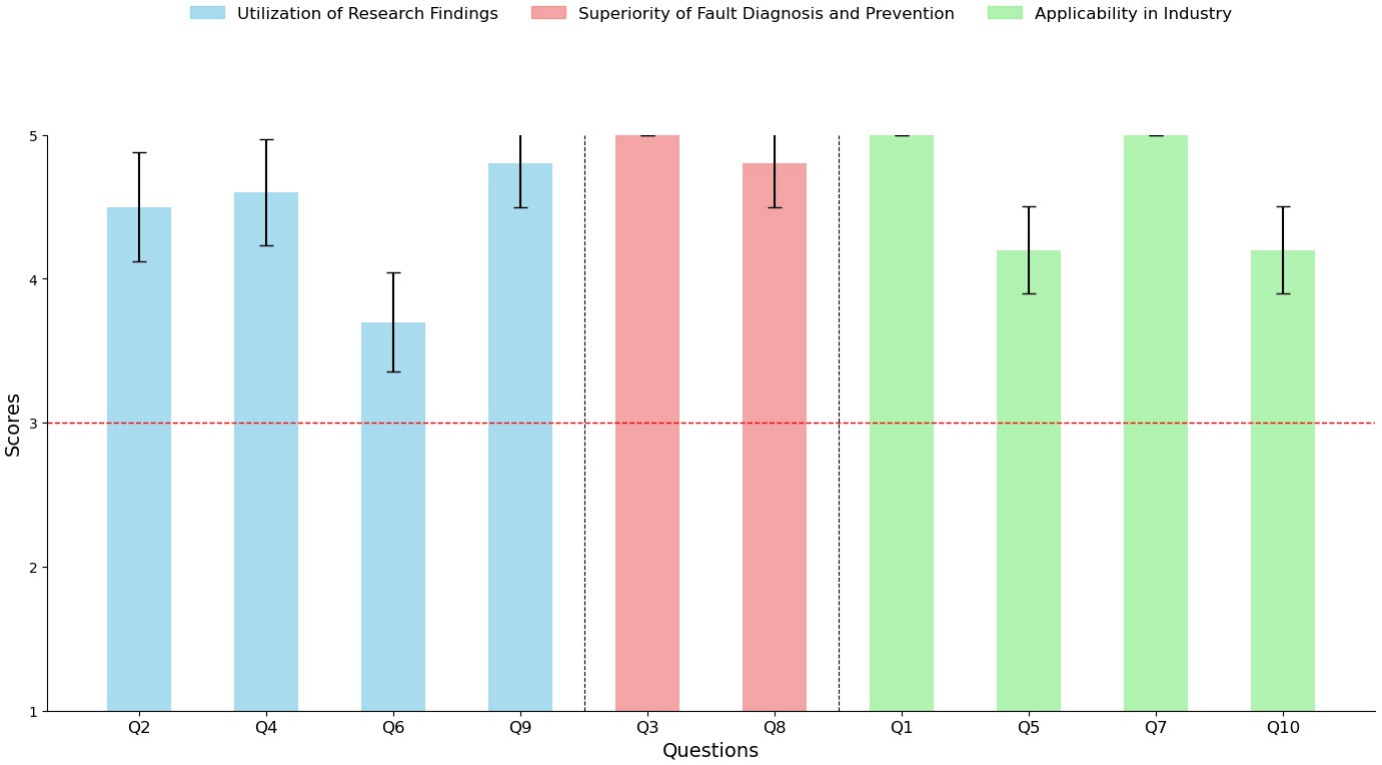
Survey results.

As illustrated in Fig 16, showing the mean and confidence intervals for each questionnaire, survey results revealed a positive tendency across all 10 items. The Wilcoxon Signed-Rank Test results indicated that all items had p-values below 0.05, signifying statistically significant differences compared to the neutral response (3 on the Likert scale). Moreover, the median responses for each item were higher than 3, confirming that respondents provided positive evaluations. In particular, most items had a p-value of 0.002, demonstrating a high level of statistical significance, suggesting that respondents strongly rated the proposed method positively. In conclusion, the analysis of response tendencies using the Wilcoxon Signed-Rank Test showed a positive trend for all items, indicating that the method has high practicality and applicability. Based on these positive evaluations, it can be inferred that the proposed method holds significant potential for real-world implementation in industrial settings and can make meaningful contributions to fault prevention and management.

## 7 Discussion and conclusions

This research aimed to enhance manufacturers’ ability to manage equipment failures through the innovative use of big data analytics and artificial intelligence. By leveraging equipment maintenance data, we successfully developed a quantitative risk classification system and extracted pivotal topics using BERTopic. These advancements facilitate a deeper understanding of failure mechanisms, enabling the formulation of comprehensive maintenance strategies and multifaceted risk analyses.

Utilizing readily available maintenance data, including metrics such as Mean Time Between Failures (MTBF), Mean Time To Repair (MTTR), Failure Intensity Rates, Cause of Failure, Failure Handling, our study showcases the potential of real-world data to inform and improve failure management processes. The application of BERTopic to distill significant insights from failure reports exemplifies the model’s versatility across various organizational contexts, not limited by company or equipment type. This approach, grounded in actual operational data, promises tailored applications to specific organizational needs, enhancing the relevance and impact of the findings.

To the best of our knowledge, publicly available datasets from actual industrial settings that contain cause of failure, failure handling and industrial engineering metrics documented by managers do not exist, and accessing such data is typically difficult. By using the dataset collected from actual industrial sites, our approach focuses on extracting key topics of failure and developing a quantitative risk classification system, allowing for tailored applications to meet specific organizational needs, thus enhancing the relevance and impact of the research findings.

The quantitative risk classification system proposed in this study demonstrates high flexibility, capable of applying both quantitative and qualitative methods depending on the industry sector, data volume and subject knowledge of managers. Since maintenance reports generated by manufacturers generally follow similar structures, this method can be applied across various equipment and manufacturing domains for risk grading. Additionally, insights derived through BERTopic can assist experts in identifying more objective and critical causes of equipment failures.

However, this study is not without its limitations. A notable constraint is the omission of equipment age from the data analysis, which obscure failure patterns attributable to wear and tear over time. The variability in failure characteristics and frequencies related to equipment lifespan necessitates age-specific maintenance approaches, suggesting an area for future enhancement of our model. Additionally, the scope of data analyzed, comprising 720 inspection records, points to the potential for deriving more robust insights from a larger dataset.

Despite these limitations, our study underscores the utility of equipment maintenance data as a valuable resource for failure management and risk assessment within manufacturing environments. The methodology presented here offers a pragmatic approach for companies to leverage existing data towards achieving more objective and efficient decision-making processes. As industries move towards the smart factory paradigm, the ability to conduct nuanced risk analysis based on comprehensive maintenance data will serve as a cornerstone for operational excellence and strategic foresight.

In conclusion, this research contributes a significant methodological framework for utilizing maintenance data in failure management and risk assessment. Future work should aim to incorporate additional data dimensions, such as equipment age, and expand the dataset to enhance the model’s predictive accuracy and applicability. Through continued refinement, this approach has the potential to become a cornerstone in the pursuit of operational efficiency and strategic maintenance planning in the era of smart manufacturing.
